# Evaluation of patients undergoing emergency surgery in a COVID-19 pandemic hospital: a cross-sectional study

**DOI:** 10.1590/1516-3180.2020.0181.R1.13052020

**Published:** 2020-07-06

**Authors:** Hilmi Bozkurt, Hüda Ümit Gür, Muzaffer Akıncı, Hogir Aslan, Çiğdem Karakullukçu, Doğan Yıldırım

**Affiliations:** I MD. General Surgeon and Gastroenterological Surgeon, and Doctoral Student of Molecular Oncology, Department of Gastrointestinal Surgery, University of Health Sciences, Haseki Research and Education Hospital, Istanbul, Turkey.; II MD. General Surgeon, Department of General Surgery, University of Health Sciences, Haseki Research and Education Hospital, Istanbul, Turkey.; III MD. General Surgeon, Department of General Surgery, University of Health Sciences, Haseki Research and Education Hospital, Istanbul, Turkey.; IV MD. General Surgery Assistant, Department of General Surgery, University of Health Sciences, Haseki Research and Education Hospital, Istanbul, Turkey; V MD. Anesthesiologist, Department of Anesthesiology and Reanimation, University of Health Sciences, Haseki Research and Education Hospital, Istanbul, Turkey.; VI MD. General Surgeon, Department of General Surgery, University of Health Sciences, Haseki Research and Education Hospital, Istanbul, Turkey.

**Keywords:** COVID-19 [supplementary concept], Coronavirus infections, General surgery, Emergency surgery, Pandemic hospital, COVID-19 pandemic, COVID-19 virus disease

## Abstract

**BACKGROUND:**

The COVID-19 pandemic is threatening healthcare systems and hospital operations on a global scale. Treatment algorithms have changed in general surgery clinics, as in other medical disciplines providing emergency services, with greater changes seen especially in pandemic hospitals.

**OBJECTIVES:**

To evaluate the follow-up of patients undergoing emergency surgery in our hospital during the COVID-19 pandemic.

**DESIGN AND SETTING:**

Cross-sectional study conducted in a tertiary-level public hospital.

**METHODS:**

The emergency surgeries carried out between March 11 and April 2, 2020, in the general surgery clinic of a tertiary-care hospital that has also taken on the functions of a pandemic hospital, were retrospectively examined.

**RESULTS:**

A total of 25 patients were included, among whom 20 were discharged without event, one remained in the surgical intensive care unit, two are under follow-up by the surgery service and two died. Upon developing postoperative fever and shortness of breath, two patients underwent thoracic computed tomography (CT), although no characteristics indicating COVID-19 were found. The discharged patients had no COVID-19 positivity at follow-up.

**CONCLUSION:**

The data that we obtained were not surgical results from patients with COVID-19 infection. They were the results from emergency surgeries on patients who were not infected with COVID-19 but were in a hospital largely dealing with the pandemic. Analysis on the cases in this study showed that both the patients with emergency surgery and the patients with COVİD infection were successfully treated, without influencing each other, through appropriate isolation measures, although managed in the same hospital. In addition, these successful results were supported by 14-day follow-up after discharge.

## INTRODUCTION

The first case of COVID-19 (new type of coronavirus, 2019-nCoV) in Turkey was identified on March 11, 2020, which was also the date on which the World Health Organization declared the outbreak to be a pandemic.^1^^,^^2^^,^^3^ As COVID-19 has spread around the world and across Turkey, the hospitals that have been used to treat the disease have become locations where there is a high risk of infection.^4^^,^^5^

Between this first case in Turkey on March 11, 2020, and a survey on April 2, 2020, a further 18,135 cases were identified. Approximately 60% of these cases were seen in the city of Istanbul where our hospital is located.^6^^,^^7^ Thus, Istanbul alone accounts for more than half of all patients among the 81 cities of this country. Moreover, the hospital in which we work was the first and is now one of the four officially declared pandemic hospitals in the city. The intensity of patient demand is higher around these hospitals, which gave us a responsibility to report our data because of the high reliability of our results.^7^

With the increasing incidence rate of the disease in our country, the number of patients diagnosed with or suspected of having COVID-19 infection entering our emergency service and inpatient services has also rapidly increased. It has therefore been recommended that elective surgeries should be postponed wherever possible, in regions to which the pandemic has spread.^8^^,^^9^

However, this is not possible with emergency surgeries, and so it has become necessary to carry out such surgeries while taking the maximum of precautions. Healthcare professionals are at increased risk of exposure to COVID-19 and its infection, given their involvement in treatment of this disease. This has brought to light the risk that the healthcare workforce for fighting against the COVID-19 pandemic may become diminished through illness. Given the way in which this disease has spread and affected healthcare systems around the world, it can be understood that hospitals must provide services for individuals who have been diagnosed with or are suspected of having COVID-19 infection, while also treating emergency surgery patients. After our institution was declared a pandemic hospital, emergency surgeries continued to be performed there, in a controlled manner. The COVID-19 pandemic is affecting healthcare systems and treatment approaches through its new and unknown features.

## OBJECTIVE

In this study, we aimed to evaluate the follow-up of patients who underwent emergency surgery in our hospital during the COVID-19 pandemic.

## METHODS

This study consisted of a cross-sectional evaluation of the emergency surgeries performed in the general surgery clinic of a tertiary- care hospital between March 11, 2020, when the first official COVID-19 case in Turkey was reported, and April 2, 2020. Elective and semi-elective surgeries were excluded from this study. The parameters examined included the patients’ demographic data, comorbidities, indications for surgery, preoperative patient assessment environment, preoperative imaging methods, infection parameters, surgical procedures, anesthetic procedures, postoperative intensive care requirement, length of hospital stay, status of postoperative COVID-19 suspicion and postoperative morbidity and mortality rates. All patients were evaluated with regard to whether there was any suspicion of or a diagnosis of COVID-19 before they were admitted to the hospital and after discharge,

### Our approaches in the process of adaptation to the pandemic

Pursuant to a decision by the COVID-19 Scientific Advisory Board at the start of the outbreak, all suspected COVID-19 cases were evaluated in isolated areas, and were diagnosed and treated in separate areas within the emergency service. Thus, patients presenting for surgery at the emergency service were considered to have been prevented from coming into contact with suspected COVID-19 patients.^8^

The initial examinations on the surgical patients were made using personal protective equipment, initially using masks and gloves. However, as the number of COVID-19 cases rapidly increased, all surgical patients were assumed to have contracted this disease, and N95 or filtering facepiece (FFP) masks, protective googles and protective gowns started to be used as routine. Patients who were scheduled for surgery and admitted to the service were taken into individual rooms. They were then moved to the operating room wearing a mask, and without spending any time in the preoperative room.

In order to avoid bringing the patient into contact with too many people prior to anesthesia, the anesthesia care team consisted only of an anesthesiologist and an anesthetic technician. Under the assumption that the patient was COVID-19 positive, the anesthetic procedures were carried out with the operative wearing an N95 protective mask with a surgical mask over it, a surgical box gown and protective googles.

Intubations were made by specialist physicians using a videolaryngoscope, with rapid induction and without mask ventilation. Complete nasal oxygenation was ensured. During wakening, oxygen was administered through a nasal cannula in order to avoid post-extubation masking. Recovery and wakening occurred in the operating room when possible, and the patient was transferred directly to the surgical service without entering the postoperative room. Patients requiring intensive care were transferred to the intensive care negative-pressure isolation room, accompanied by the anesthesiologist, via a different corridor. The same personal protective equipment was used for patients receiving spinal anesthesia. The surgical team used liquid-tight gowns, surgical masks over N95, protective googles and gloves (double), as personal protective equipment.

Postoperative visits to patients were conducted with a minimum of personnel (one physician and one nurse), wearing N95 masks and a surgical box gown upon each entry to and exit from the room, and using hand disinfectant. At the time of discharge, the patients were given the recommendation that they should adhere to a 14-day isolation period. On the 14^th^ day after discharge, these patients were called by phone, to gain information.

### Ethical approval

Board of ethics approval for this study was obtained from the ethics commission of the Health Sciences University, Haseki Training and Research Hospital, under approval number 40-2020, dated April 17, 2020.

### Statistical analysis

Descriptive statistics were calculated, including means, standard deviations, medians, minimums, maximums, frequencies and ratios. The SPSS 22.0 software was used for the analyses.

## RESULTS

This study included a total of 25 patients, of whom nine were male and 16 were female, with a mean age of 50 years. The nine male patients all had comorbidities. Table 1 shows the patients’ comorbidities, preoperative laboratory values, preoperative imaging, anesthetic procedure, duration of operation, length of hospital stay, postoperative complications and mortality. Among all the patients, six of them had more than one comorbidity, and none presented any suspicion of preoperative COVID-19 or had any history of contact.

**Table 1.** General characteristics and demographic data

Characteristics	Range (min-max)	Median	Mean ± SD or n and %
Age (years)	19.0-87.0	58	50.0 ± 22.3
Over 65 years old			9 (36%)
Sex			
Female			16 (64.0%)
Male			9 (36.0%)
Duration of operation (minutes)	35.0-180.0	60	84.8 ± 44.7
Length of hospital stay (days)	2.0-21.0	3	6.7 ± 6.8
Comorbidity			9 (36.0%)
Preoperative chest x-ray			25 (100%)
Preoperative chest CT			11 (44.0%)
Preoperative CT suspected positive for COVID-19			0 (0%)
Anesthesia type (general)			22 (88.0%)
Need for postoperative intensive care			7 (28.0%)
Postoperative intubation			6 (24.0%)
Wound infection			4 (16.0%)
Suspected postoperative COVID-19			2 (8.0%)
Postoperative COVID-19 test			2 (8.0%)
Current status:			
Intensive care unit			1 (4%)
General surgery service			2 (8%)
Postoperative mortality			2 (8%)
Discharged without problems			20 (80%)
Preoperative laboratory parameters			
Preoperative WBC	3-39	13.0	14.4 ± 8.1
Preoperative lymphocyte	1-4	1.0	1.6 ± 0.8
Preoperative neutrophil	2-37	11.0	12.9 ± 7.8
Preoperative CRP	1-265	100.0	104.9 ± 83.6
Preoperative albumin	1.9-5	3	3 ± 0.9

Min-Max = minimum-maximum; CT = computed tomography; WBC = white blood cell; CRP = C-reactive protein; SD = standard deviation.

Upon developing postoperative fever and shortness of breath, two patients underwent a thoracic computed tomography (CT), which did not reveal any imaging suggestive of COVID-19. Material was sent for COVID-19 polymerase chain reaction (PCR) tests and the results were negative. These two patients were discharged upon regression of their fever and cough.

Mortality occurred in the cases of two other patients who had undergone operations due to ischemia. During the 14-day follow-up on the patients who had been discharged, there was no suspicion of COVID-19 and findings of the disease.

Table 2 shows the operations and indications of the patients. The most common cause of admission was acute appendicitis. Three patients were operated on to treat findings of diffuse peritonitis due to leakage of an anastomosis. Three patients were operated on due to mesenteric ischemia because of late admission with acute abdominal and necrotic findings. Two patients were operated on to treat rectal malignancies due to mechanical colon obstruction. In one patient, bleeding did not stop despite endoscopic intervention to treat upper gastrointestinal system bleeding, and hemodynamic deterioration was observed. One patient was operated on to treat mechanical intestinal obstruction due to adhesions, since there was no intestinal passage despite follow-up for 72 hours. In one patient, intestinal evisceration occurred during postoperative mobilization. In one patient, retraction of the intestinal stoma into the abdomen occurred on the postoperative third day. In one patient who had undergone an inguinal hernia repair because of anticoagulant usage, widespread postoperative hematoma and active blood leakage was observed. One patient was operated on due to abundant hemorrhage following failure of endoscopic gastrostomy revision, by means of endoscopic therapeutic intervention.

**Table 2.** Patient diagnoses and types of surgery

Indication for operation	Procedures	Patient Number
Acute appendicitis	Appendectomy (open)	3
Acute appendicitis	Appendectomy (laparoscopic)	7
Anastomosis leak	Feeding jejunostomy, loop ileostomy, Hartman colostomy	3
Mesenteric ischemia	Subtotal colectomy, segmental ileum resection, diagnostic laparoscopy	3
Malignancy rectum	Sigmoid loop colostomy	2
Bleeding in the upper gastrointestinal tract	Pyloroplasty	1
Adhesive ileus	Adhesiolysis	1
Postoperative evisceration	Postoperative exploration	1
Stoma retraction	Stoma revision	1
Unreduced umbilical hernia	Hernia repair	1
Postoperative hematoma	Hematoma drainage	1
Gastrostomy obstruction	Gastrostomy revision	1
Total		25

All the patients were operated on under emergency conditions. None of the patients presented suspected COVID or any diagnostic history before hospital admission, and none of these patients had any signs or symptoms of COVID-19 over the 14-day follow-up after discharge.

## DISCUSSION

In the present study, the changes in approaches to general surgery and emergency practices resulting from the COVID-19 pandemic were examined. The pandemic continues to spread rapidly, and has affected all healthcare service units and operations around the world, and Turkey has not been an exception.

We demonstrated that necessary surgeries can still be performed in COVID-19 pandemic hospitals if the measures and precautions required are taken. Despite research into the epidemiology, pathophysiology, treatment and practices relating to patients infected with COVID-19, and the respective effects on public health, algorithms for surgical treatment on individuals with a suspicion or diagnosis of COVID-19 infection have been deemed of secondary importance.^9^ Earlier in the pandemic, anesthesiologists published various recommendations for the equipment and protection that was required to protect themselves during intubation.^8^^,^^11^^,^^12^ Subsequently, recommended approaches to surgery were suggested for patients with a suspicion or diagnosis of COVID-19.^3^^,^^5^^,^^10^

Although a considerable proportion of COVID-19 patients are asymptomatic, it is an infectious disease. Therefore, all our surgical patients in our pandemic hospital were approached under the assumption that they were positive, from the earliest days. Since the virus is mainly transmitted through droplets, it is accepted that the virus will remain on contact surfaces for hours, or even days, as a potential source of infection through transmission by contact.^11^ This constitutes a risk for healthcare professionals, who may come into contact with these contaminated surfaces, thus transmitting the virus to themselves or others.^13^ All surfaces were disinfected, and efforts were made to leave as much time as possible between two operations. Both the anesthesia care team and the surgical team tried to have the minimum number of staff present in the room during induction of anesthesia.

The use of laparoscopy in emergency surgery on patients suspected of or diagnosed with COVID-19 is a matter of controversy, given that there is still no clear information in this regard. Yu et al.^14^ reported that SARS-CoV-2 spreads through droplets and contact, although fecal-oral and aerosol transmissions cannot be ignored. They therefore stated that laparoscopic surgeries could be performed on colon cancer patients infected with COVID-19, although there would be a need for good management of laparoscopic gases. Likewise, Morris et al.^15^ suggested that the laparoscopic approach could be used for gynecological cases, and that the risk of COVID-19 had yet to be proven. In the present study, seven patients underwent laparoscopic appendectomy and one patient underwent diagnostic laparoscopy with a diagnosis of mesenteric ischemia. No problems were encountered during the postoperative follow-up on the seven patients. Considering that use of laparoscopy reduces the length of hospital stay, we conclude that its use should be preferred in emergency surgery when performed by experienced surgeons.

Some studies have recommended follow-up at home using antibiotics, with communication via telephone or other means, for cases of non-complicated acute appendicitis.^16^ Such patients, however, were scheduled for surgery, even though there was no perforation, considering that there might not have been sufficient ways means of communication and access in the pandemic area, with poor access to healthcare facilities. A total of ten patients who underwent appendectomy were discharged without event.

In a review by Di Saverio et al.,^3^ it was recommended that all patients who were to undergo surgery should be tested for COVID-19. Such tests were not requested in the earliest of our cases, given that the test results would not be available immediately, and the surgical decision would be unaffected anyway. With increasing numbers of COVID-19-positive patients and tests (PCR), we started to seek tests in new cases, as the routine. In a similar vein, no thoracic CT was requested in the earliest days, but came to be sought even if the patient was asymptomatic, in line with the latest recommendations.^5^ Thoracic CT was requested in the cases of 11 patients, along with abdominal CT (44%).

Protective measures were also taken in relation to cases operated on previously in our hospital that were admitted for surgery due to complications during the process. Such patients were considered to be at risk because of their contact with visitors and healthcare personnel. Furthermore, two patients with rectal tumors underwent emergency surgery for a temporary protective loop colostomy.

Given that all elective surgery and diagnostic endoscopy procedures have been postponed because of the extended duration of the pandemic, there is growing concern about the increasing the frequency of presentations to the emergency services with a clinical picture of ileus due to colon and rectal tumors. The limitations of the present study are the limited number of patients, the single-center design, the lack of randomization and the retrospective nature of the study.

## CONCLUSION

We believe that the risk of dissemination of COVID-19 will be reduced through isolating the emergency surgery services from the pandemic services, by means of using personal protective equipment, carrying out preoperative abdominal CT simultaneously with thoracic CT (when required) and ensuring the minimum of contact between healthcare staff and the patient. The data that we obtained were not surgical results from patients with COVID-19 infection. They were the results from emergency surgeries on patients who were not infected with COVID-19 but were in a hospital largely dealing with the pandemic. Analysis on the cases in this study showed that both the patients with emergency surgery and patients with COVİD infection were successfully treated, without influencing each other, through appropriate isolation measures, although managed in the same hospital. In addition, these successful results were supported by 14-day follow-up after discharge.
